# Prevalence of Childhood Illnesses and Care-Seeking Practices in Rural Uganda

**DOI:** 10.1100/tsw.2003.52

**Published:** 2003-08-19

**Authors:** Anthony K. Mbonye

**Affiliations:** Department of Community Health, Ministry of Health, P.O. Box 7272 Kampala, Uganda

**Keywords:** children, illness, prevalence, health care, Uganda

## Abstract

There is a declining trend of child health indicators in Uganda despite intensified program efforts to improve child care. For example, the infant mortality rate increased from 81/1,000 in 1995 to 88/1,000 in the year 2000. This paper presents results of a study that assessed factors responsible for this trend. The objectives were to assess the prevalence of childhood illnesses and care-seeking practices for children with fever, diarrhea, and upper respiratory tract infections (URTI) in the Sembabule district of Central Uganda. A cross-sectional survey, using a WHO 30 cluster-sampling technique, was used to obtain data from 300 women with children aged less than 2 years. Prevalence of childhood illnesses and care-seeking practices were obtained using a structured questionnaire supplemented by in-depth interviews. The results showed that the 300 women interviewed had 323 children of whom 37.9% had an episode of fever 2 weeks before the survey, 40.3% had diarrhea, 37.4% had URTI, and 26.8% were fully immunized. Most of the women, 82.7%, perceived fever as the most serious health problem to their children. URTI, diarrhea, and measles were perceived as serious by a lower proportion of women. Although this study showed high perceptions of childhood diseases, the proportion of mothers seeking care for sick children was low, indicating that there are barriers to accessing care. For example, 44.7% of women sought care when their children had fever, 35.0% when children had URTI, and 31.3% when children had diarrhea. However, most children with fever, diarrhea, and URTI were treated at home and taken to health units only when they developed life-threatening symptoms. This late referral to health units was complicated by high costs of care, long distances to health units, poor attitude of health workers, lack of drugs at health units, and limited involvement of fathers in care of the children.The results of this study showed that although the perceptions of childhood diseases were high, the care-seeking practices were poor. In order to improve child care in this district, there is a need to address barriers to quality of care and to conduct further research to assess the role of cultural factors and male involvement in child care.

### INTRODUCTION

Several studies have shown that people diagnose, treat, and care for sick people on the basis of how they perceive the signs and symptoms of a particular disease, the importance given to these signs and symptoms, and the understanding of the cause and the expected outcome[1]. The timely treatment of malaria is in the hands of those people who have primary responsibility for health within their families. In most communities, women are responsible for their own health, that of their children, husbands, and others[2,3,4]. Up to 90% of childhood illnesses are first perceived, defined, and treated at home — mostly by the mothers — before being referred elsewhere for treatment[5].

A recent review of studies on treatment-seeking behavior has documented that care seeking for malaria was related to cultural beliefs about the cause and care of illness. Multiple treatment options are often used, especially when the first treatment fails. This review found that a large proportion of malaria cases are treated outside the formal health care system[6].

Malaria is the leading cause of mortality and morbidity among pregnant women and children in Uganda[1,6,7]. Most areas in Uganda have perennial malaria transmission. Early diagnosis and treatment of malaria cases is one of the basic epidemiological tools of the current global malaria control strategy. This control strategy is emphasized and reflected in most country strategic plans in malaria endemic areas in sub-Saharan Africa as well as in Uganda[1,5]. Women recognize malaria as a serious disease to children in Uganda. Care seeking is mainly to drug shops (where they purchase antimalaria drugs), and only a limited number go to formal health units as a last resort if there is treatment failure[4].

Similar findings have been documented in the coastal district of Kilifi in Kenya, where most sick children were treated promptly by the purchase of over-the-counter drugs at retail outlets[7]. In this district, mothers recognized convulsions, anemia, and spleenomegarly, but did not link these to malaria. The study further found that 99.7% of mothers reported that they would seek advice elsewhere, before taking a sick child to a health facility.

A review of the literature showed that there is no previous study that has assessed perceptions on childhood diseases in Uganda. It is therefore important to conduct such a study to obtain data in order to formulate a health promotion package to improve heath-seeking behavior for sick children, and reduce the current high morbidity and mortality among children in Uganda.

This paper presents findings from a study carried out in the Sembabule district of Central Uganda in 2001. The aim of the study was to assess prevalence of childhood illnesses and care-seeking practices among women in the district. The specific objectives of the study were to estimate the prevalence of malaria, diarrhea, upper respiratory tract infections (URTI), and immunization coverage and to assess care-seeking practices for children sick from malaria, URTI, and diarrhea.

### METHODS

#### Study Area and Population

The Sembabule district lies in a remote area in Central Uganda. The district has a poor road network and is served by one health center and five dispensaries. The district is divided into five subcounties, which are different in terms of socioeconomic activities and ways of life. Mijwara subcounty is in the central area of the district and is inhabited by Baganda, who are mainly farmers and engage in some limited business activities. Lwebitakuri subcounty is in the east of the district and is inhabited by Bakiga immigrants, who are agricultural peasants. Lwemiyaga and Ntusi subcounties are in the southern and western parts of the district, respectively, and are inhabited by the Bahima and Banyarwanda, who are pastoralists. These pastoralists move from one area to another in search of grass and water for their cows, especially in the dry season. The district has a flat geographical terrain with a poor source of clean water. The main source of water in the district is from streams, ponds, and wells that provide water for home use and for the animals.

The district has two dry seasons from December to March and from May to July. The dry seasons are important in two aspects: they are when malaria transmission peaks and when there is scarcity of water, which makes it difficult to maintain proper hygiene.

### Study Design

A sample of 300 women with children aged less than 24 months was randomly selected from five subcounties of the district using the WHO 30 cluster-sampling technique. The sampling frame was obtained from the Housing and Population Census[8]. All houses in the 30 selected clusters were listed and visited by trained interviewers. All women who had children less than 24 months of age were interviewed on a range of issues affecting child health using a structured questionnaire. In-depth interviews were carried out among key informants who included community health workers, trained traditional birth attendants, teachers, and local leaders. The themes explored in these interviews included definition of local terms for diseases, perceptions of childhood illnesses, and care-seeking patterns. In total, 50 in-depth interviews were carried out.

All questionnaires were initially translated into Luganda, the local language understood by most people in the district. The questionnaires were pretested and revised. Interviewers were selected to include social workers and health workers who were trained and assisted in revision of the questionnaires.

### Case Definitions

Fever was defined as a child feeling hotter than usual, associated with loss of appetite and general weakness. In the Luganda language, the local term is “omusujja” and this term is widely known among most mothers in Uganda[4]. Diarrhea was defined as watery stools that occured at least three times a day, and URTI was defined as cough accompanied by rapid short breathing.

The period of recall of diarrhea, fever, and URTI episodes was limited to 2 weeks preceding the survey. The study elicited data on a single episode of any of the illnesses within this period.

### Data Analysis

Data were double entered using EPINFO version 6.0. Bivariate analysis was carried out to identify variables associated with diarrhea, malaria, URTI, and immunization status. Associations were tested using a Chi-square test and a $p$-value of less than 0.05 was considered significant.

Qualitative data were initially coded and analyzed manually. Since the same themes were explored by the two data sets, results from the qualitative study were used mainly in the interpretation of findings.

## RESULTS

### Sociodemographic Characteristics

A total of 300 women, aged between 15–49 years, with 323 children, were interviewed on a range of factors affecting childhood illnesses, care-seeking patterns, and where care for sick children was sought.

Most women, 84.2%, were aged 20–39 years, 13.1% were adolescent mothers aged 15–19 years, and a few, 2.7%, were older women aged 40–49 years. A large proportion of women (58.2%) had completed primary education, while 37.8% had not attained any education at all. A smaller percentage (4.0%) had attained secondary education and above.

Most women respondents (67.3%) were peasant farmers engaged in agricultural activities; 18.4% were involved in cattle keeping, selling milk and other animal products; while 14.3% of the women reported they were doing no work to generate money (see Table 1). Immunization of children was different across subcounties, with Ntusi and Lwemiyaga having low immunization levels. However, the prevalence of diarrhea, fever, and URTI was not different across subcounties. The mother's education was not associated with prevalence of diarrhea ($p > 0.43$), immunization coverage ($p > 0.22$), and prevalence of URTI ($p > 0.37$). This is because over 90% of respondents either had no education or had attained only primary education.

**TABLE 1**
**Sociodemographic Characteristics of Women Interviewed**

| | | |
|---|---|---|
| **Age structure of women respondents** | | |
| 15–19 | n = 39 | 13.1% |
| 20–39 | n = 252 | 84.2% |
| 40–49 | n = 9 | 2.7% |
| **Education level of women respondents** | | |
| No education | n = 113 | 37.8% |
| Completed primary | n = 174 | 58.2% |
| Completed secondary | n = 13 | 4.0% |
| **Occupation** | | |
| Housewife (no work) | n = 43 | 14.3% |
| Agriculture | n = 202 | 67.3% |
| Cattle keeping | n = 55 | 18.4% |

### Burden of Childhood Illnesses

Out of a total of 323 children aged less than 24 months, 37.9% had an episode of fever, 40.3% had diarrhea, and 37.4% had URTI 2 weeks before the survey. Prevalence of diarrhea was highest among children aged 6–11 months, 49.5%, and those aged 12–23 months, 37.5%. Diarrhea was also high among children 0–5 months, accounting for 34.1%. Similarly URTI was high among children 6–11 months, 46.2%, and those aged 12–23 months, 38.8%. Fever was more prevalent among children aged 12–23 months accounting for 47.7%. Immunization for children aged 12–23 months was low at 30.8% (see Table 2).

**TABLE 2**
**Prevalence of Fever, Diarrhea, URTI, and Immunization Coverage of Children Aged <24 Months**

| Age of Child (months) | Prevalence of Diarrhea | Prevalence of URTI | Immunization Status | Children with Fever |
|---|---|---|---|---|
| 0–5 | 34.1% | 26.7% | 21.9% | 25.4% |
| 6–11 | 49.5% | 46.2% | 27.7% | 40.6% |
| 12–23 | 37.5% | 38.8% | 30.8% | 47.7% |
| All Children | 40.3% | 37.4% | 26.8% | 37.9% |

### Perception of Serious Symptoms of Childhood Illnesses and How This Influences Care-Seeking Patterns

The majority of women (82.7%) perceived fever caused by mosquitoes as the most serious health problem to children. Fewer women (38.1%) perceived URTI as serious, 32.8% perceived diarrhea, 31.3% measles, and 9.0% thought skin diseases were the most serious health problems to their children.

The proportion of women who sought care when their children were sick was 44.7% for fever and this was significantly lower than the proportion of women who perceived malaria as a serious health problem, 82.7% ($\chi^2 = 31$, $p < 0.0001$).

However there was no significant difference between mothers who perceived and sought care for URTI (38.1% vs. 35.0%, $\chi^2 = 0.2$, $p > 0.66$), diarrhea (32.8% vs. 31.3%, $\chi^2 = 0.9$, $p > 0.76$), and/or mothers who perceived measles and children who received measles vaccine (31.3% vs. 31.0%, $\chi^2 = 0$, $p = 1.0$) (see Table 3).

**TABLE 3**
**What Women Perceive as the Most Serious Health Problems to Their Children and the Care-Seeking Patterns**

| Most Serious Health Problem to Education | Proportion of Women Who Perceive a Health Problem as Serious | Proportion of Women Seeking Care (when Sick or Taken for Immunization) | |
|---|---|---|---|
| Malaria | 82.7% | 44.7% | $\chi^2 = 31$, $p < 0.0001$ |
| Respiratory diseases | 38.1% | 35.0% | $\chi^2 = 0.2$, $p = 0.66$ |
| Diarrhea | 32.8% | 31.3% | $\chi^2 = 0.9$, $p = 0.76$ |
| Measles | 31.3% | 31.0%* | $\chi^2 = 0.0$, $p = 1.0$ |
| $DPT_3$ | 10.8% | 5.7%** | $\chi^2 = 1.6$, $p = 0.20$ |

* Percentage of children who received measles vaccine.
** Percentage of children who received $DPT_3$ vaccine.

### Knowledge of Signs of Disease and Care-Seeking for Children

A hypothetical question was posed to mothers as to what signs and symptoms of a disease would require them to seek immediate care for a child and this was compared with the proportion of mothers who sought immediate care when the child developed the particular sign or symptom of the disease.

Some of the mothers, 39.0%, said they would seek care when a child developed a dry mouth, sunken eyes, and decreased urine output, while 86.8% sought immediate care when their children developed the signs ($\chi^2 = 49$, $p < 0.0001$). Similarly, 20.7% said they would seek care for prolonged diarrhea, while 80.0% sought immediate care ($\chi^2 = 69$, $p < 0.0001$); 11.5% said they would seek care if a child had vomiting, while 64.3% sought immediate care ($\chi^2 = 57$, $p < 0.0001$); 16.7% said they would seek care if a child had fever, while 78.3% sought immediate care ($\chi^2 = 74$, $p < 0.0001$); 26.6% said they would seek care if a child failed to drink or breastfeed, while 88.6% sought immediate care ($\chi^2 = 78$, $p < 0.0001$); 24.7% said they would seek care if a child felt weak, while 84.4% sought immediate care for a weak child ($\chi^2 = 67$, $p < 0.0001$) (see Table 4). These results showed that the seriousness of signs and symptoms of disease is an important determinant of care-seeking pattern for a sick child.

**TABLE 4**
**Perceptions on Seriousness of Diarrhea and Care-Seeking Pattern**

| Common Symptoms of Diarrhea | Proportion of Mothers who Sought Care when a Child had One of the Symptoms | Proportion of Mothers who would Seek Care when a Child had Severe Symptoms | |
|---|---|---|---|
| Dry mouth, sunken eyes, decreased urine output | 86.8% | 39.0% | $\chi^2 = 49$, $p < 0.0001$ |
| Prolonged diarrhea | 80.0% | 20.7% | $\chi^2 = 69$, $p < 0.0001$ |
| Vomiting | 64.3% | 11.5% | $\chi^2 = 57$, $p < 0.0001$ |
| Fever | 78.3% | 16.7% | $\chi^2 = 74$, $p < 0.0001$ |
| Failure to drink or breastfeed | 88.6% | 26.6% | $\chi^2 = 78$, $p < 0.0001$ |
| Weakness | 84.4% | 24.7% | $\chi^2 = 67$, $p < 0.0001$ |

### People Seeking Care for Sick Children

Overall, mothers were responsible for seeking care for 33.1% of children who had diarrhea, 33.8% who had fever, 47.6% who had URTI, and 52.9% of the children who were taken for immunization.

Fathers were responsible for care seeking for 17.3% of the children who had diarrhea, 21.4% who had fever, 34.5% with URTI, and 35.3% of the children who were taken for immunization. Older children were responsible for taking care of 20.4% of the children who had diarrhea, 17.9% with fever, 16.7% with URTI, and 28.4% took children for immunization.

Relatives were the most frequent child caregivers after the mothers. Relatives were responsible for taking care of 27.6% of children with diarrhea, 24.8% with fever, 38% with URTI, and 40.7% took children for immunization (see Table 5).

**TABLE 5**
**People Seeking Care for Children Aged <24 Months Suffering from Malaria, Diarrhea, or URTI**

| Person Seeking Care for Sick Child | Child with Diarrhea | Child with Malaria | Child with URTI | Child Taken for Immunization | Average |
|---|---|---|---|---|---|
| Mother | 33.1% | 33.8% | 47.6% | 52.9% | 41.9% |
| Relative | 27.6% | 24.8% | 38.0% | 40.7% | 32.8% |
| Father | 17.3% | 21.4% | 34.5% | 35.3% | 27.1% |
| Older child | 20.4% | 17.9% | 16.7% | 28.4% | 20.9% |
| House girl/maid | 0.8% | 1.4% | 1.2% | 1.3% | 1.2% |
| Neighbor | 0.8% | 0.7% | 1.4% | 12.3% | 3.8% |

### Places where Care was Sought when Children were Sick

Most care for fever (75%) was obtained from drug shops, 9.2% of the mothers sought care from health units, 5.1% from private clinics, 6.1% from a community health worker, and 4.1% from a traditional healer. Contrarily, 49.5% of mothers sought care from health units when children had diarrhea, while 15.8% sought care from drug shops. Other mothers, 11.9%, sought care from a traditional healer or used traditional herbs (see Table 6).

**TABLE 6**
**Places Where Care for Sick Children was Sought**

| Place Where Care was Sought | Child Sick with Malaria | Child Sick with Diarrhea |
|---|---|---|
| Drug shops | 75.5% | 15.8% |
| Health units | 9.2% | 49.5% |
| Private clinic s | 5.1% | 31.7% |
| Community health worker | 6.1% | 5.0% |
| Traditional healer/herbs | 4.1% | 11.9% |
| Traditional birth attendant | — | 4.0% |
| Relative | 6% | 14.0% |

### Mothers Perceptions of Ways to Prevent Malaria

Few mothers knew how malaria could be prevented. For example, only 10.5% knew that sleeping under a mosquito net could prevent malaria, 15.8% knew about cutting bushes around houses, and 13.5% knew that reducing stagnant water around homes reduced the breeding sites for mosquitoes. Similarly only 10.5% of the mothers knew that spraying houses with insecticides was helpful in reducing mosquitoes.

In addition to this low knowledge on ways of preventing malaria, 16.0% of respondents thought that malaria could never be prevented, while 19.5% did not know any method of preventing malaria.

### DISCUSSION

The results of this study showed that there is high morbidity due to malaria, diarrhea, and URTI among children in the Sembabule district of Uganda. Although this study showed that there was high perceptions of childhood diseases, the proportion of mothers seeking care for sick children was low, indicating that there are barriers to accessing care. Most children with fever, diarrhea, and URTI were treated at home and only taken to health units when they developed life-threatening symptoms. This late referral to health units was complicated by high costs of care, long distances to health units, poor attitude of health workers, lack of drugs at health units, and limited involvement of fathers in care of the children.

These results were consistent with findings from the Uganda Demographic Health Survey–2000[9], which reported high morbidity and mortality among children in Uganda. In this survey, 44% of children aged less than 5 years had fever, 20% had diarrhea, 23% had a cough accompanied by rapid breathing, and 37% of the children were fully immunized[9]. The results further indicated that the proportion of children suffering from diarrhea who were taken to a health worker declined from 50.6% in 1995 to 29.1% in 2000. Similarly the proportion of children suffering from URTI who were taken to a health unit declined from 36.4 to 32.3% in the same period[9].

Although Integrated Management of Childhood Illnesses (IMCI) was established in 1995 in Uganda to assist in reducing high morbidity among children, this involved the training of health workers with little community involvement. A study carried out in the year 2000 found that there was poor referral of children to health units, where only 30% of children who needed referral actually were. The following factors were identified for the poor referral: high costs, bad attitudes of health workers, unavailability of transport, and poor quality of care[8].

In this study (see Table 2), we found a high prevalence of diarrhea among children aged 0–5 months. This is probably due to the weaning practices in this area. From in-depth analysis, it was found that most children at that age were given cow's milk at weaning. This practice was common in the two subcounties of Ntusi and Lwemiyaga. (These are the subcounties with many cows in the district.) There is, therefore, a need to educate mothers on exclusive breast feeding for 4 months as recommended by the policy in Uganda[10].

We found that the proportion of mothers seeking health care for sick children was low. This does not correlate well with the proportion of mothers who perceived illnesses among children. This study has identified some factors that affect care seeking such as lack of awareness, poor quality of care at health units, and social cultural factors that were elicited during in-depth interviews. Further to this, the hypothetical question "would you seek care if a child had any of the following signs" gives misleading data on care seeking. This renders hypothetical questions of little value in health-seeking behavior studies. McCombie earlier arrived at a similar conclusion in a review of treatment seeking for malaria[6].

In addition, there is disparity seen between the proportion of mothers who sought care from health units when their children had fever, 9.2%, and those who sought care when their children had diarrhea, 49.5% (see Table 6). This is because diarrhea leads to overt signs like weakness and sunken eyes that caretakers easily recognize as serious symptoms, whereas most fevers in children occur as mild to moderate and a child with fever can play and eat food. The disparity also can be explained by the current health messages on radio and television on how to treat malaria in adults and children using chloroquine and Fansidar, which are easily obtained from drug shops. In addition, there are initiatives in Uganda to implement a program on home management of malaria, and this could lead to people thinking they do not need to go to health units for treatment. There is a need to evaluate how this strategy influences treatment seeking for malaria in Uganda and other countries where implementation of this strategy is taking place.

The study further showed that mothers were the major players in seeking care for children. An additional important finding was that fathers, older children, and relatives seek care for a significant proportion of sick children including taking them for immunization services. This demonstrated the importance of the family in health care seeking, but ways must be sought for more involvement of fathers in child care.

Most care for children suffering from fever was sought from drug shops. The reason for this is because drug shops in Uganda are widespread and easily accessible. However, a recent study carried out in Uganda has shown that only 34% of prescriptions at drug shops follow the national recommended guidelines, putting into doubt how effective this care outlet is in managing malaria[6]. The results concur with findings by Kengeya et al.[4], which showed that for most women the first action for fever was to buy tablets from nearby shops. If this treatment fails they will take the patient to health units.

The results of this study further showed that about 12% of children with malaria were treated by traditional healers or using traditional herbs. This finding was consistent with findings of Kengeya et al.[4]. Both studies were carried out in two neighboring districts, although at different times, and showed that this practice could be widespread in most districts of Uganda.

The knowledge of how malaria can be prevented was low in this district, thus posing a challenge to intensified health education as a means of strengthening malaria control in the Sembabule district. Results from this study partly differ from findings from the Kilifi district in Kenya, where childhood malaria was perceived as mild and an everyday illness[2]. This study however found low knowledge among mothers on malaria prevention and the care-seeking patterns. Most antimalaria drugs for children sick with the disease were purchased over the counter at retail shops. Results from both studies indicate that it is important to develop messages using a simple language understood by the mothers on the dangers of these diseases if care is not sought.

Morbidity due to fever among children was measured using the definition “omusujja” — translated as fever — and was perceived by a majority of women as a serious health problem to their children. The definition had a limitation, because a number of childhood diseases like URTI, measles, and diarrhea may present with fever. Thus this term is not specific and it affects the categorization of childhood diseases used in this study. This notwithstanding, in highly malaria-endemic areas, malaria is the leading cause of fevers and presumptive diagnosis is always used since laboratory facilities are inadequate.

### CONCLUSIONS

The results of this study have the following implications: In order to improve the high disease burden among children in this district, there is a need to target communities so that care-seeking practices for sick children are improved. This would involve redesigning IMCI programs to include a community component to sensitize child caretakers on danger signs and appropriate care. A referral system involving communities is currently being piloted in Uganda and results are being awaited[11]. There is also a need to intensify health education messages on ways of preventing malaria and other childhood diseases, explaining the importance of seeking care at health units and targeting fathers and other members of the extended families in child care practices. These measures could reduce child mortality and morbidity in this district of Uganda. Further research is suggested to identify the role of culture factors and male involvement in child care.

### BIOSKETCH

**Anthony K. Mbonye, MD, MA, MPH**, is currently the Acting Assistant Commissioner of Health Services at the Department of Community Health, Ministry of Health, Kampala, Uganda. His current research interest is testing new approaches in prevention of malaria in pregnancy at the community level.
